# Exploring consumer adoption of smart sportswear through an integrated acceptance model based on perceived comfort

**DOI:** 10.1038/s41598-025-19809-7

**Published:** 2025-10-14

**Authors:** Mengyun Yang, Mi Luo, Jiabing Jin, Jia Zhang

**Affiliations:** 1 Faculty of Creative Arts and Design, UCSI University, Kuala Lumpur, Malaysia; 2 School of Fashion Design, Jiangxi Institute of Fashion Technology, Nanchang, China; 3 School of Textile Science and Engineering, Jiangnan University, Wuxi, China; 4 School of Physical Education, Chongqing University, Chongqing, China

**Keywords:** Smart sportswear, Consumer acceptance, UTAUT2, VAM, Perceived comfort, Psychology, Human behaviour

## Abstract

With the enhancement of people’s health awareness and the rapid development of smart sportswear technology, it is of great significance to study smart sportswear from the perspective of consumers. This study aims to explore the factors influencing consumers’ acceptance of smart sportswear by constructing a comprehensive model. The model integrates the Extended Unified Theory of Acceptance and Use of Technology (UTAUT2) and the Value-Based Adoption Model (VAM), with perceived comfort as a key factor. We collected a total of 486 valid samples and employed Structural Equation Modeling (SEM) to analyze the data. The results indicate that both UTAUT2-related factors (hedonic motivation (HM), effort expectancy (EE), social influence (SI), and facilitating conditions (FC)) and comfort-related factors (perceived comfort (PC), material comfort (MC), ergonomic design (ED), and technology integration comfort (TIC)) have significant direct or indirect effects on consumers’ adoption intention (IA). In contrast, performance expectancy (PE) and perceived cost (PCo) do not significantly impact perceived value (PV). This study highlights the unique psychological mechanisms underlying the adoption of smart sportswear, underscores the critical role of comfort in shaping consumer intention, and extends existing technology acceptance models by incorporating multidimensional comfort constructs. These findings offer novel theoretical insights and provide practical guidance for user-centered design and future development of smart sportswear.

**Supplementary Information:**

The online version contains supplementary material available at 10.1038/s41598-025-19809-7.

## Introduction

With the increasing demand for wearable devices, flexible wearable technology (FWT) has become an important research area^1^. Wearable technologies encompass clothing and accessories that integrate electronic components or utilize smart textiles^2^. This trend includes flexible electronic devices such as health monitors, sensors, displays, and portable energy storage, all of which have experienced rapid growth^3^. While smart wearable technology is evolving, existing research does not provide a full examination of the consumer psychology of smart sportswear, particularly when contrasted to traditional clothing. This study gap is most visible in the limited investigation of consumer acceptance, adoption intention, and comfort. Filling this gap is critical for both theoretical research and practical applications.

With improving living standards and diverse lifestyles, the public’s health awareness has changed significantly. Sports activities have become a popular trend among the general public, driving the demand for fitness-related products and making the sportswear market increasingly segmented, professionalized, and intelligent, particularly in smart sportswear^4^. As a convergence of multidisciplinary technologies, smart sportswear has significant market potential amidst changing lifestyles^5^. In sportswear, wearable textiles not only enhance athlete comfort and protection for athletes, but also improve sports performance by integrating chemical sensors that analyze sweat components in real time^6^ .

Current research on smart sportswear mainly focuses on smart materials and functional design. Jiayi Fu et al.^7^ demonstrated the advancement of sensor technology by integrating liquid crystal color-changing core-sheath composite fibers as flexible temperature sensors in smart sportswear. Ying-Chia Huang et al.^8^ designed smart sportswear for individuals with Down syndrome, while Jia Wu et al.^9^ created customized smart clothing for sit-skiers to monitor health data and reduce injury risk. Park J. and Kim J.^10^ incorporated conductive yarn into sportswear to control LED lighting, enhancing safety for nighttime athletes.

However, few studies have explored smart sportswear from a consumer perspective. Research in traditional clothing has addressed consumer behavior, including attractiveness factors^11^eco-friendly consumption^12^cultural values^13^and materialism^14^. Yet, smart sportswear differs substantially in terms of technology acceptance, body perception, and functionality expectations. Additional concerns such as continuous body data collection, privacy, and long-term wear comfort further influence consumer motivation and adoption intention^15^.

This study is guided by the following research question: What factors drive consumers’ willingness to adopt smart sportswear in the context of a healthy lifestyle? To investigate this question, the study develops a comprehensive research model that, for the first time, incorporates perceived comfort into the Unified Theory of Acceptance and Use of Technology (UTAUT) and the Value-based Adoption Model (VAM) frameworks. Structural Equation Modeling (SEM) is employed to examine the relationships among key variables, providing both theoretical innovation and practical guidance for user-oriented design and functional optimization of smart sportswear.

The remainder of this paper is structured as follows: “Methods” presents the theoretical model and hypothesis development. “Data acquisition” introduces the data acquisition process, including questionnaire design, study design, and procedure. “Results and analysis” presents the results and analysis. “Discussion and conclusion” discusses the findings, followed by theoretical and practical implications, and outlines the study’s limitations and suggests directions for future research.

## Methods

### Research model

Venkatesh et al.^15^ extensively analyzed the previous main models (TRA, TPB, DTPB, TAM, IDT, SCT, MM and MPCU) and proposed the UTAUT. The theory holds that the four key constructs of performance expectation, effort expectation, social influence and facilitating conditions are the antecedents of behavioral intention and use behavior. While the UTAUT model has been widely used to explain technology adoption, it has certain limitations, particularly in mandatory and organizational contexts. To overcome these limitations, Venkatesh et al.^16^ introduced hedonic motivation, price value, and habit as additional constructs, transforming the UTAUT from an organizational to a customer perspective. This expanded version, known as UTAUT2, includes performance expectancy, effort expectancy, social influence, facilitating conditions, hedonic motivation, price value, and habit. Among the various models for technology acceptance, UTAUT2 stands out as the most thorough in explaining consumer acceptance and use of technology^17^. The study found that the UTAUT2 theory is substantially more predictable than the UTAT theory, accounting for around 74% of consumer behavioral intentions for focused technology and 52% of consumer technology use^18^.

Kim et al. introduced the VAM, which emphasizes perceived value by identifying benefits and sacrifices^19^. This model regards practicality and enjoyment as the main benefits, while technical complexity and perceived cost are regarded as sacrifices. It also studies how these factors affect the intention to use the technology. VAM explains the reasons for the adoption of new technologies from the perspective of value maximization, and the perceived value of using new technologies is a key predictor of intention to use^20^.

By integrating different models or extending the original model, the accuracy of prediction technology acceptance and user behavior can be significantly improved^15^. This study combines UTAUT2 and VAM frameworks to develop a comprehensive model for smart sportswear, and identifies perceived comfort (PC) as a key factor. The UTAUT2 model covers a wide range of factors that affect the use of technology, and the VAM model complements the dimension of perceived value, which makes the evaluation of user acceptance more comprehensive. This model integration ensures that the research is closer to the real consumer environment. Comfort in sportswear is an important factor in consumer choice^21^. In the analysis of expert analysts, it is generally believed that comfort plays a key role in determining the wide acceptance of clothing, so it is selected as an important factor in the model. Intelligent sportswear not only needs to have technical functions, but also needs to ensure the wearer’s comfortable experience during exercise. When users choose smart sportswear, comfort is one of the primary considerations, as it directly affects their athletic performance and overall experience.

**Fig. 1:**
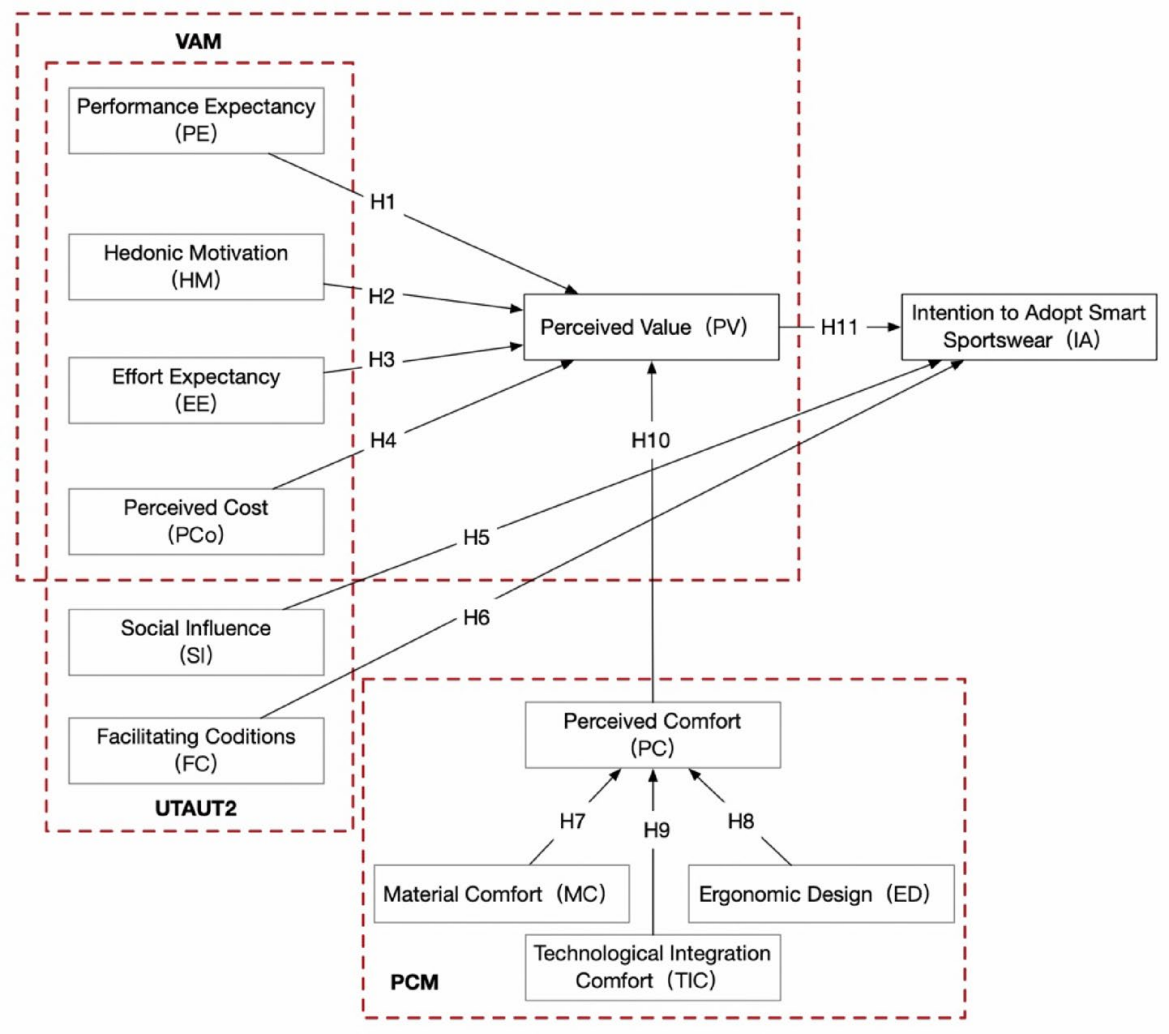
Research model.

In order to determine the sub-dimension of perceived comfort (PC), we conducted several rounds of discussions and interviews with experts in the field of smart sportswear. First of all, several experts with deep background in clothing design, sports science and technology integration were invited to participate in the brainstorming meeting. In the process, a variety of dimensions that may affect comfort were proposed. A preliminary screening was conducted to determine the key factors that are most likely to affect the user‘s perceived comfort. Subsequently, we conducted one-on-one in-depth interviews to explore the specific meaning of each factor and its importance in the actual scene. Finally, through extensive analysis and multiple rounds of feedback and revision, material comfort (MC), ergonomic design (ED) and technology integration comfort (TIC) were finally determined as the three sub-dimensions of perceived comfort. This model integrates multiple perspectives, fully considers the various interests and expectations of consumers, and enhances the practicality and relevance of research results. The model structure is presented in Fig. 1.

### Hypothesis development

#### Performance expectancy (PE)

The term “performance expectancy” relates to the amount to which users are expected to gain from the usage of technology while carrying out a specific task^15,16^. PE has been considered the most powerful factor in predicting behavioral intentions^15^. Smart sportswear can help consumers acquire body data, develop training plans, and improve their athletic performance, which can be regarded as the performance expectations of the device. Performance expectancy and perceived usefulness are critical factors in user technology acceptance behavior across various fields, such as mobile TV and the Internet of Things^12,17^. This leads to the following hypothesis:

H1: Performance expectancy of smart sportswear will positively influence users’ perceived value.

#### Hedonic motivation (HM)

Hedonic motivation is the pleasure or enjoyment derived from employing a technique, which has been shown to determine the individual’s technical acceptance^16,22^. According to Venkatesh et al., hedonic motivation, also known as perceived enjoyment, has a direct impact on individuals’ intentions to adopt technologies in different contexts^16^. In the environment of health-focused smart wearable technologies, enjoyment is critical to long-term engagement. For example, Yang et al.^23^ discovered that reported enjoyment greatly increases the perceived value of smart fitness trackers. Building on this, Ilhan and Fietkiewicz demonstrated that gamified features in fitness apps not only increase user enjoyment but also inspire long-term engagement in healthier lifestyles^24^. Therefore, the following hypothesis is proposed:

H2: Hedonic motivation will positively influence users’ perceived value.

#### Effort expectancy (EE)

Venkatesh et al. define effort expectancy as the ease with which consumers can interact with technology^16^. In the context of smart sportswear, EE assesses consumers’ impressions of the usability of smart clothes. The performance data of smart sportswear is typically shown on other electronic devices, such as smartphones. When users believe intelligent technology is simple to use, they are more likely to explore its benefits and interact with it^25^. Therefore, this study proposes the following hypothesis:

H3: Effort expectancy of smart sportswear will positively influence users’ perceived value.

#### Perceived cost (PCo)

The VAM is particularly relevant in this context, as pricing strategies can have a major impact on the market penetration of innovative products^20^. As a relatively new product, smart sportswear may trigger varied consumer responses depending on the pricing strategies employed. For instance, Niknejad et al. found that high perceived cost negatively affected the adoption of smart health wearables^26^. Similarly, Liao et al. confirmed that cost concerns reduce consumers’ perceived value in the context of smart devices^27^. Thus, the following hypothesis is proposed:

H4: Perceived cost of smart sportswear will negatively influence users’ perceived value.

#### Social influence (SI)

Social influence is defined as a change in one’s thoughts, feelings, attitudes, or behaviors as a result of interaction with another person or group^28^; in other words, being influenced by others. In the early stage of new technology adoption, social impact plays a significant positive role^29^. The opinions of influential people in people’s daily lives have a positive impact on their behavior^26^. With the development of online communities, people are increasingly relying on others’ comments and recommendations when deciding to adopt new products. Specifically, in health-related wearable technology, social encouragement from peers, fitness groups, or influencers can greatly enhance users’ willingness to adopt such products^30^. Furthermore, studies have shown that community support and online fitness communities can boost motivation for continued use of smart wearables^31^. Therefore, the following hypothesis is proposed:

H5: Social influence will positively impact consumers’ intention to adopt smart sportswear.

#### Facilitating conditions (FC)

Venkatesh et al. state that facilitation consists of a combination of extrinsic and intrinsic factors^16^. Extrinsic factors refer to an individual’s perception of the adequacy of the resources required to accomplish a particular activity, while intrinsic factors reflect an individual’s self-assessment of his or her ability to perform the activity. External conditions typically include technical support, infrastructure, and other necessary system configurations that are critical to ensuring that users can smoothly use smart sportswear. Internal conditions include users’ skill level, knowledge base, and personal confidence, which directly influence their ability and efficiency in using the new technology. Furthermore, Venkatesh et al. found that facilitative conditions can have a statistically significant impact on people’s behavioral intentions to adopt new technologies^15^. Therefore, the following hypothesis is proposed:

H6: Facilitating conditions will positively influence consumers’ intention to adopt smart sportswear.

#### Material comfort (MC)

The comfort of sportswear is greatly influenced by the material properties and their interaction with physiological responses during physical activity^32^. Material comfort includes factors such as fabric softness, moisture-wicking breathability and allergenic potential. Materials that are suitable for different climatic conditions and that maintain optimal skin temperature and humidity are essential for improving the overall comfort of users and their willingness to continue using the product. Therefore, the following hypothesis is proposed:

H7: Higher material comfort ratings will positively influence users’ perceived comfort.

#### Ergonomic design (ED)

Ergonomic design aims to align human needs with design elements to enhance comfort, health, safety, functionality, and aesthetics, moving beyond traditional design concepts focused on visual appeal and efficiency^33^. Ergonomic design of smart sportswear focuses on how the garment fits and supports the body during various physical activities. It involves designing for a wide range of motion without restricting flexibility, incorporating adaptive fit, stretchability, and strategic placement of seams and padding. Ergonomic design can prevent discomfort and potential injury while increasing the overall functionality and user satisfaction of smart sportswear. Therefore, the following hypothesis is proposed:

H8: The ergonomic design of smart sportswear will positively influence users’ perceived comfort.

#### Technological integration comfort (TIC)

Technological integration comfort evaluates how comfortably technology components are integrated into sportswear. This includes evaluating the placement of sensors and devices to ensure they do not impede movement or cause discomfort during physical activities. Sensors can be categorized into flexible and rigid types. Flexible sensors, made of stretchable materials, retain their properties, while non-flexible sensors, made of brittle materials, are more rigid and are usually silicon-based. Despite their broad application, non-flexible sensors have drawbacks such as stiffness and instability^34^. Integration should be seamless, making users barely aware of the sensors during activities. This factor is crucial as intrusive technology may deter users from adopting smart sportswear despite its functionality. Therefore, the following hypothesis is proposed:

H9: Technological integration comfort of smart sportswear will positively influence users’ perceived comfort.

#### Perceived comfort (PC)

Comfort is significantly influenced by garment selection, which affects both the physiological and psychological well-being of the wearer. Understanding this dynamic is essential for consumer satisfaction and critical for manufacturers. Factors such as fibers, yarns, fabrics, and finishing processes all influence comfort and the success of the final product^35^. The comfort perspective is essential to understanding the adoption of smart sportswear, as the user’s physical interaction with the product can significantly influence its acceptance. This section examines three key components of comfort: material comfort, ergonomic design, and technological integration comfort. Therefore, the following hypothesis is proposed:

H10: Perceived comfort of smart sportswear will positively influence users’ perceived value.

#### Perceived value (PV)

The perceived value has become a significant factor in the widespread adoption of intelligent technologies^20^. Perceived value not only affects consumer preferences and evaluations of products, but also influences consumer choices^36^. For smart sportswear, perceived value may stem from functionality, durability, comfort, and how these attributes enhance fitness habits and overall health management. As awareness of health and fitness increases, the value proposition of smart sportswear becomes increasingly important. Therefore, the following hypothesis is proposed:

H11: The higher perceived value of smart sportswear will positively influence consumers’ adoption intentions.

Based on the prior explanation, Fig. 1 depicts the hypothesized relationships between all variables. To test this model, we carried out a survey-based study as described below.

## Data acquisition

This study employed quantitative research methodologies and collected data through an anonymous online questionnaire. It was conducted in accordance with the Declaration of Helsinki, and approved by Jiangxi Institute of Fashion Technology. Informed consent was obtained from all individual participants involved in the study.

### Questionnaire design

To validate the suggested study model, an online questionnaire was created that included all relevant constructs. The majority of the measuring items were modified from past relevant research and adjusted to the context of smart sportswear. The new scale primarily includes items related to perceptions of smart sportswear and intentions to adopt smart sportswear, which accurately reflect the specific context of use. All items were scored on a 7-point Likert scale, with 1 (strongly disagree) to 7 (strongly agree)^37^. To ensure response accuracy, attention check items were added in the questionnaire, such as: “Please respond ‘disagree’ to this item to confirm that you have carefully read all the questions”^38^.

To make the research topic more intuitive, a randomly selected smart sportswear product available on the market was used. Participants were provided with both a written description and visual images of the smart sportswear to stimulate their perceptions^39^ and enable them to gain a more concrete understanding of the product. The images of the smart sportswear are shown in Table 1. After reviewing the images and text, participants were required to complete the survey.

**Table 1:**
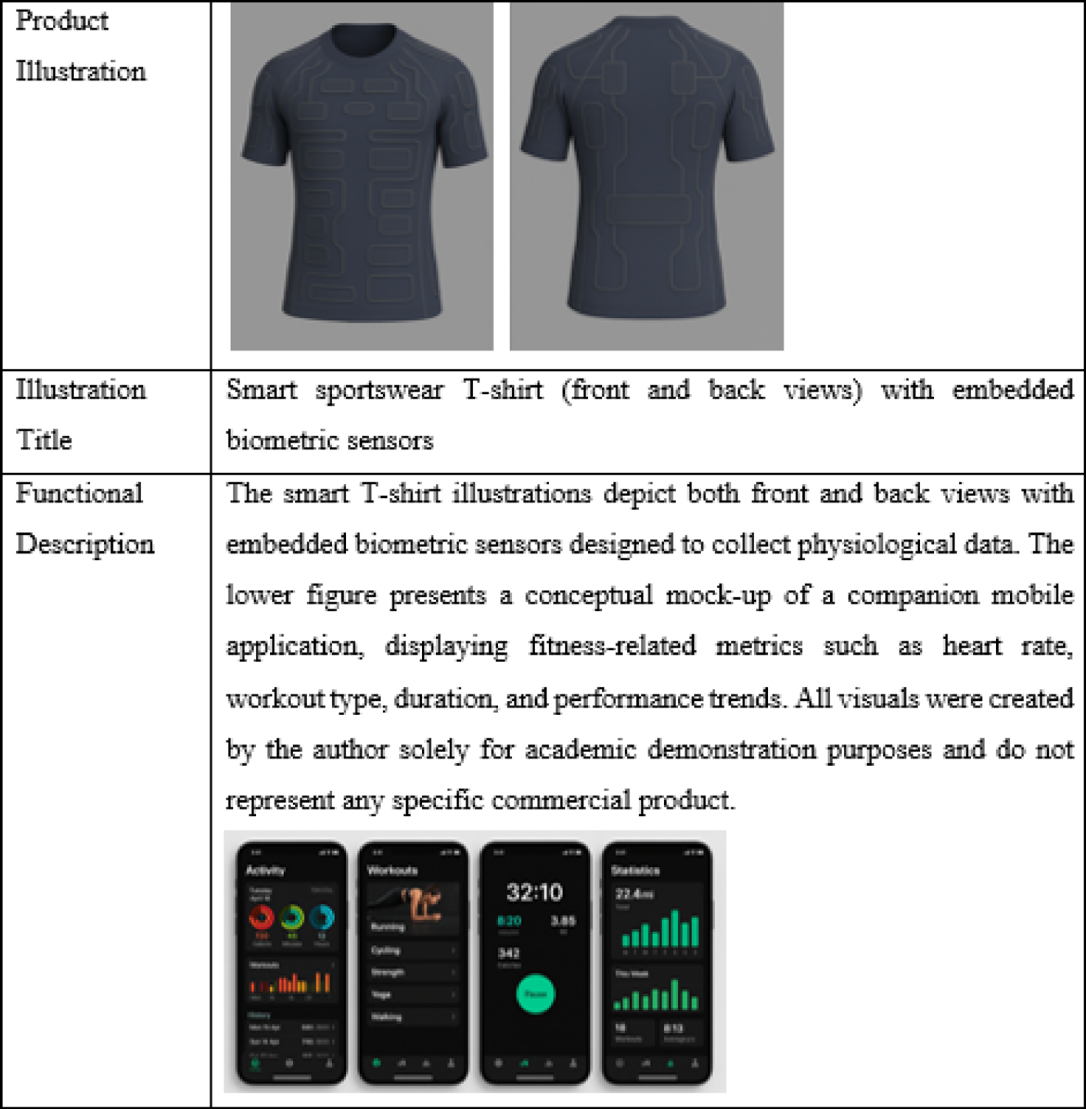
Conceptual example of smart sportswear and its corresponding mobile app interface.

In addition, three professional researchers and experts in technology acceptance theory were invited to review the questionnaire for logical consistency, appropriate use of terminology, content relevance, and question clarity. A pilot study with 30 students was conducted to gather feedback and refine the questionnaire. Based on preliminary research and expert recommendations, further adjustments were made to the format, question order, and clarity of wording.

### Study design and procedure

The survey consisted of two parts: the first part collected respondents’ demographic information, such as age, gender, educational background, and experience with smart wearable devices; the second part involved constructs related to the proposed research model. The questionnaire was distributed via Wenjuanxing, a widely used online survey platform in China, which enabled efficient data collection and management. Participants were recruited between May and June 2024 through multiple WeChat-based channels, including posts in fitness-related group chats, WeChat Moments (social timeline), and direct invitations from researchers and volunteers. This approach was chosen to effectively reach the study’s target population—individuals who frequently exercise in gyms, outdoor venues, or parks, and who are interested in smart sportswear or wearable fitness devices. Screening questions were included to confirm that respondents had some familiarity or experience with such products.

No monetary or material incentives were provided for participation. The recruitment process relied on voluntary response via social media networks, which may introduce some degree of self-selection bias. While this sampling approach is non-random, it allowed the researchers to focus on a health-conscious, tech-aware demographic aligned with the research objective. As such, the sample may not be fully representative of the general population, but it reflects the characteristics of a relevant target user group for smart sportswear adoption.

The data were initially processed using Python 3.8.6 for preliminary data cleaning and exploratory analysis. Confirmatory Factor Analysis (CFA) and Structural Equation Modeling (SEM) were subsequently conducted using covariance-based SEM (CB-SEM) with Mplus 8.1, which provided the model fit indices reported in the Results section. Due to issues of multicollinearity among the observed variables and low factor loadings, two rounds of Confirmatory Factor Analysis (CFA) were conducted, resulting in adjustments in the relevant sections of the questionnaire. These adjustments were based on expert feedback and further literature review to ensure that the revised items could more accurately measure the relevant constructs. Appendix A provides the updated measurement items used in the second-round survey, including all constructs and corresponding sources. The revised questionnaire was redistributed and a second survey was conducted, yielding a total of 534 responses. After rigorous screening to eliminate extreme outliers and obviously careless or incomplete responses, 486 questionnaires were retained for subsequent data analysis.

Table 2 shows the respondents’ demographic distribution, which reflects current social trends in technology adoption and health awareness. A larger proportion of male respondents (59.26%) reported interest in or access to smart sportswear, which may be attributed to gendered differences in fitness behavior and technology engagement—men are more likely to adopt wearable fitness devices and actively participate in sports activities, particularly in Chinese urban environments. Most participants were between the ages of 21 and 39, with young adults (21–29 years, 46.09%) and middle-aged adults (30–39 years, 37.85%) making up the majority. This distribution is consistent with the ideal consumer base for smart sportswear items, since these age groups are more health-conscious, technologically savvy, and financially independent, making them more likely to invest in smart fitness solutions.In terms of physical exercise patterns, more than half of respondents (54.73%) reported exercising for 1–2 h per session, while 68.41% reported exercising 5–10 times per month. This high level of physical activity reflects China’s growing fitness culture, which includes gym memberships, outdoor running, and body tracking.

These data illustrate the features of the sample’s target group as well as the potential market demand for smart sportswear for actual use. Understanding these demographic characteristics enables the research to develop targeted product development, thereby increasing market acceptance and user satisfaction.

**Table 2:** Demographic profile of the respondents (*n* = 486).

Variable	Description	Frequency	Percentage
Gender	Male	288	59.26%
	Female	198	40.74%
Age	Under 20	78	16.05%
	21–29	224	46.09%
	30–39	109	37.85%
	40–49	53	26.77%
	More than 50	22	4.53%
Profession	Employee	286	58.85%
	Student	67	13.79%
	Household	101	20.78%
	Retiree	14	2.88%
Exercise duration	Under 1 h	154	31.69%
	1–2 h	266	54.73%
	More than 2h	66	13.58%
Exercise frequency (per month)	Less than 4 times	51	10.49%
	5–10 times	332	68.41%
	More than 10 times	103	21.19%

## Results and analysis

### Common method variance test

As all of the variables in this study were measured using the participants’ self-report, it was necessary to test their common method variance^40^. Harman’s single-factor method was used to assess whether common method bias existed^41^. An exploratory factor analysis (without rotation) including all of the questionnaire items found that the first factor explained 29.998% of the total variance, which is well below the critical threshold of 40%, suggesting that CMV is unlikely to pose a serious threat to the validity of the results.

Additionally, a confirmatory factor analysis (CFA) was conducted using the CB-SEM approach, specifying a single-factor model in which all measurement items loaded onto a common latent factor. The model fit indices (*χ²/df* = 6.12, CFI = 0.668, TLI = 0.684, RMSEA = 0.165) were not satisfactory, further suggesting that common method bias did not pose a serious concern in this sthdy^40^.

### Measurement model

The measurement model was evaluated by assessing internal reliability and convergent validity standards^42^. Cronbach’s α and composite reliability (CR) were used to assess the reliability of the constructs. For internal reliability, Cronbach’s α values and CR for each construct should exceed 0.70, with CR at least 0.6 and average variance extracted (AVE) no less than 0.6^43^. Meanwhile, the standardized factor loadings of the measurement items on their respective constructs ranged from 0.727 to 0.928, which were all significantly higher than the recommended value of 0.70, indicating that the measures can better reflect the latent variables to which they belong. In this study, Cronbach’s *α* values ranged from 0.873 to 0.936 (Table 3), indicating strong internal reliability. Additionally, the KMO value was 0.871, indicating that the data are suitable for factor analysis.

**Table 3:** The reliability of the measurement model.

Constructs	Items	Loading	Cronbach’s α	CR	AVE
Performance expectancy	PE1	0.901	0.936	0.934	0.825
	PE2	0.903			
	PE3	0.893			
Hedonic motivation	HM1	0.880	0.928	0.927	0.810
	HM2	0.894			
	HM3	0.926			
Perceived cost	PCo1	0.880	0.923	0.921	0.796
	PCo2	0.876			
	PCo3	0.919			
Effort expectancy	EE1	0.876	0.915	0.917	0.786
	EE2	0.926			
	EE3	0.855			
Social influence	SI1	0.917	0.914	0.917	0.787
	SI2	0.921			
	SI3	0.820			
Facilitating conditions	FC1	0.914	0.911	0.911	0.773
	FC2	0.873			
	FC3	0.849			
Perceived value	PV1	0.927	0.910	0.910	0.772
	PV2	0.793			
	PV3	0.909			
Perceived comfort	PC1	0.851	0.907	0.907	0.764
	PC2	0.895			
	PC3	0.875			
Material comfort	MC1	0.854	0.904	0.904	0.758
	MC2	0.907			
	MC3	0.848			
Ergonomic design	ED1	0.794	0.895	0.895	0.738
	ED2	0.928			
	ED3	0.850			
Technological integration comfort	TIC1	0.954	0.887	0.892	0.736
	TIC2	0.876			
	TIC3	0.727			
Intention to adopt	IA1	0.818	0.873	0.872	0.694
	IA2	0.837			
	IA3	0.844			

To assess discriminant validity among the latent constructs, the Fornell–Larcker criterion was applied. As shown in Table 4, the square root of the AVE for each construct (bold values on the diagonal) is greater than its correlations with any other construct, indicating satisfactory discriminant validity^44^. Although Fornell–Larcker is traditionally used in PLS-SEM, it was applied here for its robustness, despite the use of CB-SEM.

**Table 4:** Discriminant validity table.

	PE	HM	PCo	EE	SI	FC	PV	PC	MC	ED	TIC	IA
PE	**0.908**											
HM	0.227	**0.900**										
PCo	0.424	0.299	**0.892**									
EE	0.364	0.355	0.292	**0.887**								
SI	0.323	0.312	0.391	0.307	**0.892**							
FC	0.327	0.387	0.299	0.357	0.213	**0.879**						
PV	0.308	0.385	0.371	0.400	0.343	0.380	**0.879**					
PC	0.365	0.271	0.410	0.275	0.256	0.309	0.336	**0.874**				
MC	0.341	0.325	0.338	0.338	0.372	0.292	0.379	0.285	**0.871**			
ED	0.380	0.300	0.406	0.426	0.328	0.354	0.357	0.317	0.370	**0.859**		
TIC	0.413	0.288	0.333	0.380	0.391	0.303	0.288	0.283	0.418	0.327	**0.877**	
IA	0.405	0.392	0.375	0.427	0.395	0.325	0.354	0.373	0.404	0.462	0.385	**0.833**

### Structural model and hypothesis testing

**Fig. 2:**
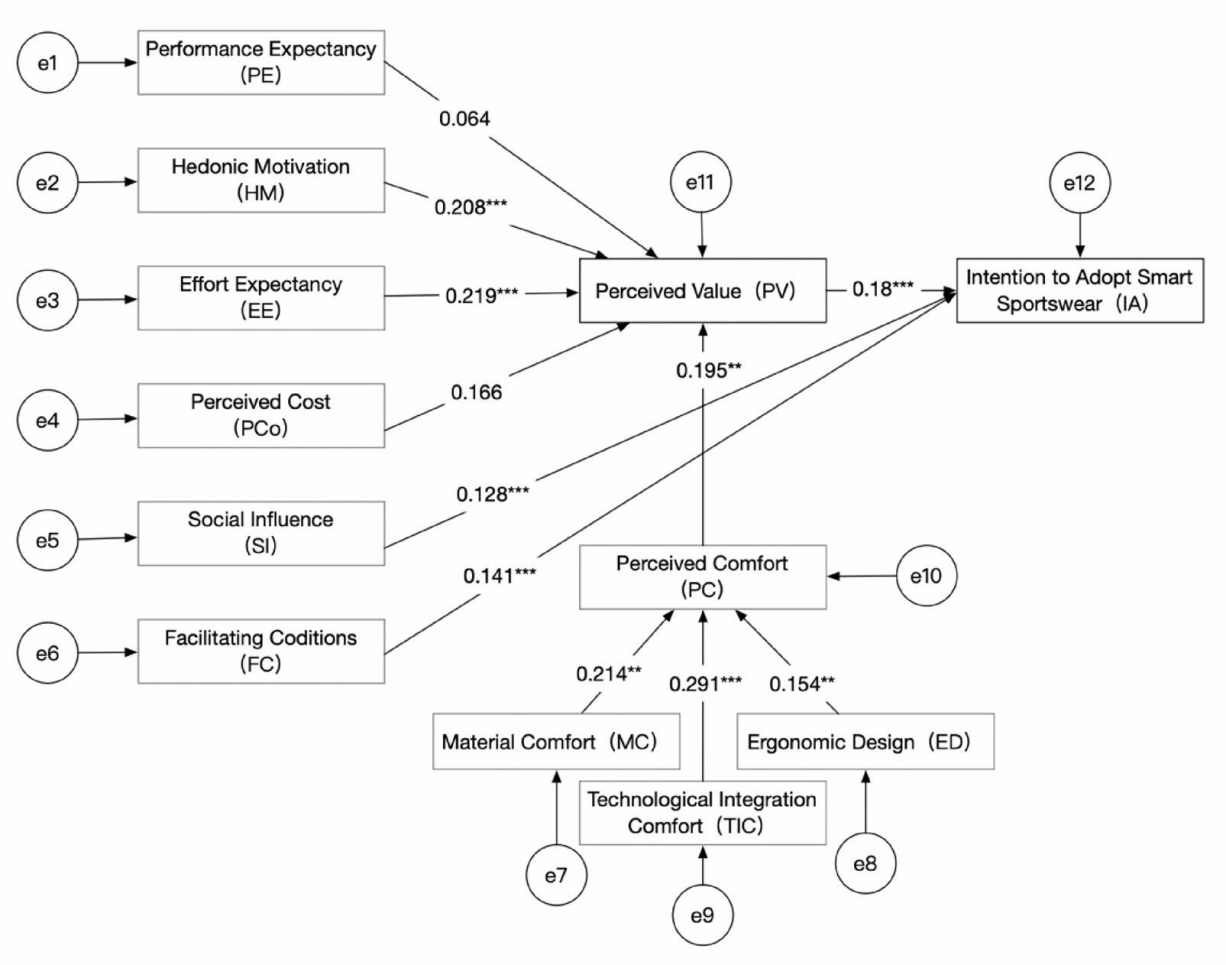
Path analysis. ***p < 0.001; **p < 0.01; *p < 0.05.

The structural model was tested using CB-SEM. Figure 2 illustrates the standardized path coefficients, while Table 5 summarizes the path coefficients, significance levels, and hypothesis testing results. The results indicate that HM and EE significantly influence PV, supporting hypotheses H2 and H3. SI and FC significantly affected IA, supporting hypotheses H5 and H6. In addition, MC, ED, and TIC significantly influenced PC, supporting hypotheses H7 to H9. PC significantly influenced PV (H10), and PV further significantly affected IA, supporting hypothesis H11.

However, H1 and H4 were not supported, indicating that PE and PCo did not significantly influence PV. The non-significant relationship between PE and PV may suggest that consumers prioritize comfort, ease of use, and social influence over performance when evaluating smart sportswear. Similarly, the lack of a significant effect of PCo may indicate that cost is not a decisive factor in perceived value among the target population, possibly because they place greater emphasis on comfort and usability.

**Table 5:** Structural model results and hypothesis testing.

Hypothesis	Path	β	Supported
H1	PE → PV	0.064	No
H2	HM → PV	0.208***	Yes
H3	EE → PV	0.219***	Yes
H4	PCo → PV	0.166	No
H5	SI → IA	0.128***	Yes
H6	FC → IA	0.141***	Yes
H7	MC → PC	0.214**	Yes
H8	ED → PC	0.154**	Yes
H9	TIC → PC	0.219***	Yes
H10	PC → PV	0.195***	Yes
H11	PV → IA	0.180***	Yes

***p < 0.001; **p < 0.01; *p < 0.05.

## Discussion and conclusion

### Discussion

This study proposed and empirically validated a comprehensive model integrating UTAUT2, VAM, and perceived comfort to explore the key factors influencing consumers’ acceptance of smart sportswear. The results indicate that HM, EE, and SI significantly affect PV, while PE and PCo do not. Additionally, MC, ED, and TIC significantly impact PC, which subsequently contributes to higher perceived value and stronger adoption intention. These findings highlight the importance of user experience—particularly comfort and usability—as a stronger determinant of acceptance than performance or cost expectations.

The results confirm that HM and EE significantly affect PV, supporting hypotheses H2 and H3. When smart sportswear is enjoyable to use and easy to use, users are more likely to perceive its value. SI and FC significantly affect IA, supporting hypotheses H5 and H6, indicating that social approval and facilitating conditions are important for users’ adoption intentions. MC, TIC, and ED significantly influence PC, supporting hypotheses H7-H9. This highlights the critical role of comfort in the user experience. When selecting and using smart sportswear, users prioritize comfort, which includes the feel of the fabric, seamless integration of technology, and rational design. PC significantly affects Perceived Value (PV), supporting hypothesis H10, indicating that cost perception significantly influences users’ perceived value. Furthermore, PV significantly affects IA, supporting hypothesis H11. This suggests that users’ perception of the value of smart sportswear positively influences their intention to adopt it.

PE did not significantly affect PV (H1), suggesting that users may not prioritize performance benefits, possibly due to unclear needs or overshadowing factors such as enjoyment and ease of use. In addition, while hypothesis H4 proposed that PCo would have a significant negative impact on PV, the results show that although the path coefficient for PCo is positive, its impact is not significant. This suggests that cost may not significantly increase perceived value. Users may choose not to use smart sportswear because of high perceived cost or because they focus more on the comfort and functionality of the product than on its price.

In today’s societal context of increasing health awareness and widespread adoption of wearable technology, the demand for smart sportswear is growing. However, when consumers make purchase decisions, in addition to considering the technical performance of the product, they will also consider the experience and perceived value, such as whether it is comfortable to wear and whether the price is reasonable. Therefore, manufacturers need to take these factors into account when designing and promoting smart sportswear to improve user satisfaction and market acceptance.

### Theoretical implications

This study provides a number of important theoretical advances to the literature on technology acceptability and smart wearables:

1. By combining constructs from the UTAUT2 and VAM with the first presented construct of perceived comfort, this study shows a comprehensive and original framework for explaining user acceptance of smart sportswear. This integration provides a more complete knowledge of the cognitive, emotional, and experiential factors influencing adoption behavior.
2. Conceptualizing and operationalizing perceived comfort in wearable technology research. This research is among the first to systematically conceptualize and operationalize perceived comfort into three distinct dimensions—material comfort, ergonomic design, and technological integration comfort—within the context of wearable sports technology. This framework adds to the existing literature by improving our comprehension of how physiological and psychological comfort variables impact technology adoption.
3. Emphasizing the central importance of perceived value in smart sportswear adoption. This study confirms that perceived value is an important concept that incorporates numerous relevant factors—such as hedonic motivation, effort expectancy, social influence, and perceived comfort—in determining consumer intention to adopt smart sportswear. These findings support the theoretical strength of the VAM and demonstrate its applicability in the context of physical wearable goods.

### Practical implications

The research results provide corresponding practical opinions for the development and design of intelligent sportswear, and have practical significance for manufacturers and marketers of intelligent sportswear. In order to increase market acceptance, manufacturers can attract users by improving technical performance and entertainment value. Specifically, there is a need to find a balance between technological innovation and cost control in order to provide more cost-effective products. Simplifying the use process can lower the user’s learning curve, increasing the product’s ease of use and attractiveness. Focusing on the comfort of materials, ergonomic design and ease of technology integration is the key to ensuring user satisfaction and increasing frequency of use. This means that these factors need to be considered at the product design stage to ensure that smart sportswear is practical and comfortable, thereby increasing user stickiness. In addition, through media publicity and celebrity endorsements, the use of social influence to promote smart sportswear, to ensure that potential users understand the advantages of the product and have the necessary resources and knowledge to correctly use these products. In addition, creating user guides and tutorials that are easy to understand and operate can help users better grasp the use of products, thereby improving user confidence and satisfaction.

### Limitations and future research

This study’s limitations including the possibility of sample bias due to certain areas and demographics. Different regions and more diverse populations should be covered in future studies to improve external validity. Although the UTAUT2 and VAM models were integrated into this study and perceived comfort was included, other factors such as privacy concerns and brand influence were not considered. In future studies, more potential factors influencing user acceptance should be explored, and qualitative methods should be incorporated to gain deeper insights.

## Supplementary Information

Below is the link to the electronic supplementary material.

Supplementary Material 1

## Data Availability

The datasets used and/or analyzed during the current study available from the corresponding author on reasonable request.

## References

[1] Li, Y. et al. Fiber-shaped asymmetric supercapacitors with ultrahigh energy density for flexible/wearable energy storage. *J. Mater. Chem. A*. **4**, 17704–17710 (2016).

[2] Ferraro, V. Smart textiles and wearable technologies for sportswear: A design approach. In *Proceedings of 2nd International Electronic Conference on Sensors and Applications* S3005 (2015).

[3] Xue, Q. et al. Recent progress on flexible and wearable supercapacitors. *Small* **13**, 1701827 (2017). 10.1002/smll.201701827 28941073

[4] Cheng, P., Wang, J., Zeng, X., Bruniaux, P. & Tao, X. Intelligent research on wearing comfort of tight sportswear during exercise. *J. Ind. Text.* **51**, 5145S–5168S (2022).

[5] Jianjian, K., Dahui, Z. H. U., Weifeng, H., Lixi, D. & Wenjing, K. Research status of intelligent sportswear. *Wool Textile Journal*. **51**, 1 (2023)

[6] Memarian, F., Rahmani, S., Yousefzadeh, M. & Latifi, M. Wearable Technologies in Sportswear. *Materials in Sports Equipment*. 123–160 (Woodhead Publishing, 2019).

[7] Fu, J., Liu, T., Yan, T. & Pan, Z. Transparent core-sheath composite fibers as flexible temperature sensor based on liquid crystal color change for smart sportswear. *J. Mol. Liq.* **393**, 123574 (2024).

[8] Huang, Y.C., Chen, J. H., Chen, G.-Y. & Tung, K. F. Smart Sportswear Design for Down Syndrome Patients. *Int. Conf. Appl. Hum. Factors Ergon*. 847–855 (Springer International Publishing, Cham, 2020).

[9] Wu, J., Kim, J. H. & Zhao, L. Exploring Smart Sportswear for Sit Skiers-Human-Centered Design Approach. *Proc. Int. Conf. Hum.-Comput. Interact*. 655–663 (Springer Nature Switzerland, Cham, 2023).

[10] Park, J. & Kim, J. The development of fitted sports wear for safety and protection using conductive yarn embroidery. *J. Fashion Bus.* **23**, 156–169 (2019).

[11] Liu, H., Guo, C. & Zhang, B. Attractiveness consumption, personality traits and sustainability: construction and empirical application of evaluation indicators for attractive attributes of China-chic T-shirt products. *Front. Psychol.* **13**, 1101978 (2022). 36643704 10.3389/fpsyg.2022.1101978 PMC9838771

[12] Gwozdz, W., Steensen Nielsen, K. & Müller, T. An environmental perspective on clothing consumption: consumer segments and their behavioral patterns. *Sustainability* **9**, 762 (2017).

[13] Patwary, S. Consumer clothing behavior and associated environmental impact. 10.20944/preprints201909.0143.v1 (2019).

[14] Hourigan, S. R. & Bougoure, U. S. Towards a better understanding of fashion clothing involvement. *Australasian Mark. J.* **20**, 127–135 (2012).

[15] Venkatesh, V., Morris, M. G., Davis, G. B. & Davis, F. D. User acceptance of information technology: toward a unified view. *MIS Q.* **27**, 425 (2003).

[16] Venkatesh, V., James, Y. L. & Xu, T. X. Consumer acceptance and use of information technology: extending the unified theory of acceptance and use of technology. *MIS Q.* **36**, 157 (2012).

[17] Wong, C. H., Han Tan, W., Loke, G., Ooi, K. B. & S.-P. & Mobile TV: a new form of entertainment? *Ind. Manag. Data Syst.* **114**, 1050–1067 (2014).

[18] Venkatesh, V., Thong, J. & Xu, X. Unified theory of acceptance and use of technology: A synthesis and the road ahead. *JAIS* **17**, 328–376 (2016).

[19] Kim, Y., Park, Y. & Choi, J. A study on the adoption of IoT smart home service: using Value-based adoption model. *Total Qual. Manag. Bus. Excell*. **28**, 9–10 (2017).

[20] Kim, H. W., Chan, H. C. & Gupta, S. Value-based adoption of mobile internet: an empirical investigation. *Decis. Support Syst.* **43**, 111–126 (2007).

[21] Wilfling, J., Havenith, G., Raccuglia, M. & Hodder, S. Can you see the feel? The absence of tactile cues in clothing e-commerce impairs consumer decision making. *Int. J. Fashion Des. Technol. Educ.* **16**, 224–233 (2023).

[22] Brown, S. A. & Venkatesh, V. A. Model of adoption of technology in the household: A baseline model test and extension incorporating household life cycle. *Manag. Inform. Syst. Q.* **29**, 11 (2005).

[23] Yang, J. Designing a technology mash-up to support remote physical-digital fashion design collaboration [PhD thesis]. Brisbane: The University of Queensland, School of Information Technology and Electrical Engineering (2014).

[24] Ilhan, A. & Fietkiewicz, K. J. Learning for a Healthier Lifestyle Through Gamification: A Case Study of Fitness Tracker Applications. *Perspectives on Wearable Enhanced Learning (WELL) Curr. Trends Res. Pract. *333–364 (Springer International Publishing, Cham, 2019).

[25] Balaji, M. S. & Roy, S. K. Value co-creation with internet of things technology in the retail industry. *J. Mark. Manag.* **33**, 7–31 (2017).

[26] Niknejad, N., Hussin, A. R. C., Ghani, I. & Ganjouei, F. A. A confirmatory factor analysis of the behavioral intention to use smart wellness wearables in Malaysia. *Univ. Access. Inf. Soc.* **19**, 633–653 (2020).

[27] Liao, Y. K., Wu, W. Y., Le, T. Q. & Phung, T. T. T. The integration of the technology acceptance model and Value-Based adoption model to study the adoption of E-Learning: the moderating role of e-WOM. *Sustainability* **14**, 815 (2022).

[28] Rashotte, L. Social influence. In *The Blackwell Encyclopedia of Sociology* (Wiley, 2007).

[29] Teo, T. S. H. & Pok, S. H. Adoption of WAP-enabled mobile phones among internet users. *Omega* **31**, 483–498 (2003).

[30] Chuah, S. H. W. et al. Wearable technologies: the role of usefulness and visibility in smartwatch adoption. *Comput. Hum. Behav.* **65**, 276–284 (2016).

[31] Liang, S. et al. Construction path of smart medical system for the aged in community based on internet. In *3rd International Symposium on Smart and Healthy Cities (ISHC)*, vol. 2021, 52–56 (2021).

[32] Wei, H. T., Chan, W. S. & Chow, D. H. Systematic review of selecting comfortable sportswear: predicting wearing comfort based on physiological responses and materials properties. *Text. Res. J.* **93**, 3926–3941 (2023).

[33] Yang, X. Application of clothing ergonomics in fashion design. In *Proceedings of the International Conference on Arts, Design and Contemporary Education* (Atlantis Press, 2016).

[34] Nag, A., Mukhopadhyay, S. C. & Kosel, J. Wearable flexible sensors: A review. *IEEE Sens. J.* **17**, 3949–3960 (2017).

[35] Lavanya, S. Clothing comfort-physiological status and psychological status. *Int. J. Mod. Trends Sci. Technol.* **6**, 61–67 (2020).

[36] Woodruff, R. B. Customer value: the next source for competitive advantage. *J. Acad. Mark. Sci.* **25**, 139–153 (1997).

[37] Premkumar, G. & Ramamurthy, K. The role of interorganizational and organizational factors on the decision mode for adoption of interorganizational systems*. * Premkumar 1995 Decisi. Sci. Wiley Online Libr.* **26**, 303–306 (1995).

[38] Dahling, J. J. & Lauricella, T. K. Linking job design to subjective career success: A test of self-determination theory. *J. Career Assess.* **25**, 371–388 (2017).

[39] Nam, C. & Lee, Y. A. Validation of the wearable acceptability range scale for smart apparel. *Fashion Text*. **7**, 7–17 (2020).

[40] Podsakoff, P. M., MacKenzie, S. B., Lee, J. Y. & Podsakoff, N. P. Common method biases in behavioral research: A critical review of the literature and recommended remedies. *J. Appl. Psychol.* **88**, 879–903 (2003). 14516251 10.1037/0021-9010.88.5.879

[41] Fuller, C. M., Simmering, M. J., Atinc, G., Atinc, Y. & Babin, B. J. Common methods variance detection in business research. *J. Bus. Res.* **69**, 3192–3198 (2016).

[42] Leguina, A. A primer on partial least squares structural equation modeling (PLS-SEM). *Int. J. Res. Method Educ.* **38**, 220–221 (2015).

[43] Hair, J. F., Sarstedt, M., Ringle, C. M. & Mena, J. A. An assessment of the use of partial least squares structural equation modeling in marketing research. *J. Acad. Mark. Sci.* **40**, 414–433 (2012).

[44] Fornell, C. & Larcker, D. F. Evaluating structural equation models with unobservable variables and measurement error. *J. Mark. Res.* **18**, 39–50 (1981).

