# Supplementary material for: Exploring consumer adoption of smart sportswear through an integrated acceptance model based on perceived comfort
Source: Sci Rep. 2025 Oct 14;15:35878. doi: 10.1038/s41598-025-19809-7 (PMC12521342; doi:10.1038/s41598-025-19809-7)
Supplement: Supplementary file 1 — Supplementary Material 1 [file 41598_2025_19809_MOESM1_ESM.docx]

# Appendix A: Measurement Items

| **Constructs** | **Items** | **Source** |
| --- | --- | --- |
| Performance expectancy  (PE) | PE1: I find smart sportswear useful in my daily life.  PE2: Using SS can improve the quality of my exercise.  PE3: Using SS daily allows me to complete tasks faster. | Maillet et al. (2015) |
| Hedonic motivation  (HM) | HM1: I find using smart sportswear fun.  HM2: I find using smart sportswear enjoyable.  HM3: I find using smart sportswear entertaining. | Venkatesh et al.  (2012), |
| Perceived fee  (PF) | PF1: I find the fees associated with using SS too high.  PF2: I find the fees associated with using SS unreasonable.  PF3: Using SS brings financial risk due to higher maintenance and repair costs. | Yang et al.  (2016) |
| Effort expectancy (EE) | EE1: I find it easy to learn how to use SS.  EE2: I find SS easy to use.  EE3: I find it easy to become proficient in using SS.  EE4: I find it easy to use SS. | Gao et al. (2015), Venkatesh et al. (2012) |
| Social influence  (SI) | SI1: People important to me think I should use SS.  SI2: Influential people think I should use SS.  SI3: If my friends use SS, I will also use them. | Gao et al. (2015), Venkatesh et al. (2012) |
| Facilitating conditions (FC) | FC1: I have the resources necessary to use SS (e.g., smartphone, internet).  FC2: I have the necessary knowledge to use SS.  FC3: SS is compatible with other tools and technologies I use. | Gao et al. (2015), Venkatesh et al. (2012) |
| Perceived Value  (PV) | PV1: Compared to the effort required, using smart sportswear is beneficial for me.  PV2: Compared to the time spent, using smart sportswear is worth it.  PV3: Considering all pros and cons, I will use smart health wearables. | Venkatesh et al. (2012) |
| Perceived comfort  (PC) | PC1: I feel comfortable wearing SS during exercise.  PC2: The comfort of SS significantly influences my decision to use SS.  PC3: The perceived comfort of SS increases my usage frequency. | (Self-developed) |
| Material comfort  (MC) | MC1: The comfort of smart sportswear fabric influences my decision to use SS.  MC2: The comfort of smart sportswear fabric influences my usage frequency.  MC3: The comfort of smart sportswear fabric influences my exercise performance. | (Self-developed) |
| Ergonomic design  (ED) | ED1: The ergonomic design of smart sportswear does not hinder my movements.  ED2: The cutting of smart sportswear suits various body movements.  ED3: I can perform various sports activities freely while wearing smart sportswear. | (Self-developed) |
| Technological integration comfort  (TIC) | TIC1: I believe that the integrated technological devices in smart sportswear will not interfere with my activities.  TIC2: I believe that when I wear smart sportswear, I can barely feel the presence of technology.  TIC3: I find the technological components of smart sportswear are well-placed and cause no discomfort. | (Self-developed) |
| Intention to adopt smart sportswear  (IA) | IA1: I will try to use SS in my daily life.  IA2: I am willing to recommend SS to others.  IA3: If SS becomes commercialized, I plan to purchase it. | Venkatesh et al. (2012),  Niknejad, N. et al.（2020） |
